# Investigating the *In Vivo* Performance of Tannic Acid-Modified siRNA in the Heart and Liver

**DOI:** 10.1021/acs.bioconjchem.6c00122

**Published:** 2026-05-27

**Authors:** O. G. Hayes, J. Rädler, E. Filipiak, T. Czapik, Y. Huang, M. Ojansivu, O. Saher, M. Bood, R. Zain, S. El Andaloussi, M. Honcharenko

**Affiliations:** † Division of Biomolecular and Cellular Medicine, Department of Laboratory Medicine, 367281 Karolinska Institutet, Huddinge 14152, Stockholm Sweden; ‡ Department of Cellular Therapy and Allogeneic Stem Cell Transplantation (CAST), 214436 Karolinska University Hospital, 14186 Stockholm, Sweden; § Karolinska ATMP (Advanced Therapy Medicinal Products) Center, 367281 Karolinska Institutet, 14152 Stockholm, Sweden; ∥ Center for Rare Diseases, Clinical Genetics and Genomics, Karolinska University Hospital, SE-17176 Stockholm, Sweden; ⊥ Nucleic Acid Therapeutics, Oligonucleotides and Targeted Delivery, Discovery Sciences, AstraZeneca, 43153 Mölndal, Sweden

## Abstract

Effective extrahepatic activity of therapeutic oligonucleotides remains an unresolved and significant problem in the development of new genetic medicines. Targeted delivery to the heart is of particular interest due to the unmet need to treat an increasing health burden of cardiovascular diseases and the identification of potential genetic therapeutic targets. Drawing inspiration from literature, we investigate the potential of tannic acid (TA) modifications to improve the activity of a model siRNA sequence in the heart. We developed the synthesis of a series of conjugates containing 1–3 (TA) units to study how molecularly defined numbers of TA ligands influence activity. While we observe significant knockdown of the siRNA target gene in the liver, we did not in the heart, suggesting that although TA ligands confer improved function of siRNA in the liver, extrahepatic activity was not enhanced. In addition, hetero-bifunctional siRNA conjugates containing both TA and albumin binding motifs were designed and their synergistic effects explored. Similarly, *in vivo* data suggests that TA ligands do not increase cardiac activity of siRNA in this context; however, we can conclude that both C16 and AlbuTag ligands do significantly improve siRNA activity in the heart. We believe this work provides important bioconjugation insight for polyphenolic molecules, like tannic acid, as well as the synthesis of hetero-bifunctional siRNA conjugates generally. Moreover, our findings will inform the design of novel targeting ligands and nanomaterials, especially for molecules that may possess emergent properties when present in high multiplicity compared to their monomeric forms.

## Graphical abstract

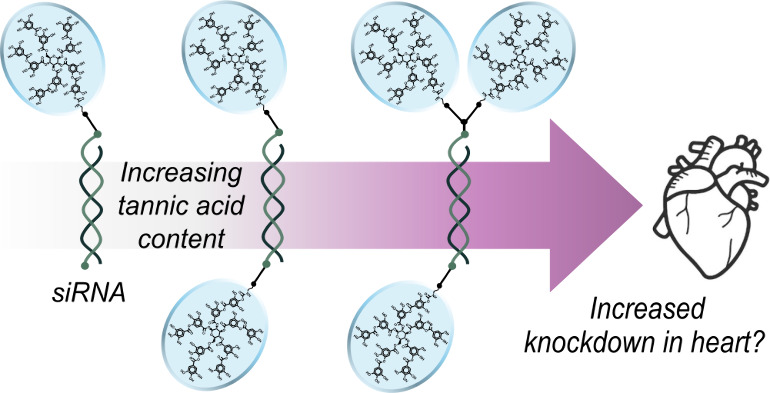

## Introduction

Oligonucleotide-based therapeutics, such as antisense oligonucleotides (ASOs) and small interfering RNA (siRNA), are critical in the development of targeted gene therapy.^1,2^ Yet, their use *in vivo*, and thus translation to the clinic, is limited by inherently low cellular uptake, a consequence of their charge, hydrophilicity and rapid degradation and clearance.^3^ Substitution of the native phosphodiester (PO) backbone for phosphorothioate (PS) and the incorporation of fluoro (F) or methoxy (OMe) modifications at the 2′ position on the ribose ring significantly increases the nuclease stability of RNA, largely solving the degradation issue.^4^ In addition, conjugation of siRNA with multiple N-acetylgalactosamine (GalNAc) units dramatically enhances delivery to liver hepatocytes.^5^ However, realizing efficient, extrahepatic delivery of these molecules still represents a significant barrier to enabling their full potential and translational success.^6^ To this end, diverse strategies have been explored to alter the delivery profile of these therapies, such as the design of oligonucleotide containing nanostructures (LNPs, liposomes, virus-like particles (VLPs), etc.)^7–11^ and chemical modification of oligonucleotides with small molecules, peptides and antibodies.^12–14^ Importantly, the conjugation of targeting ligands to oligonucleotides is an especially attractive approach as it typically yields stable, homogeneous molecules with well-defined composition and a unique tissue distribution compared to nanoparticles.^15–17^ Moreover, it has been shown that subtle changes to chemical identity^18^ and structure^19^ of such bioconjugates can significantly impact the biodistribution and efficacy of oligonucleotide therapies, indicating a vast chemical design space that can be systematically explored.

A key target organ for therapeutic genetic manipulation is the heart. In response to a steady increase in both the aging population and chronic cardiovascular disease worldwide, new therapies that can be administered noninvasively are necessary. Furthermore, important targets for gene therapy have already been identified for the treatment of diverse cardiac-involved diseases.^20,21^ In the event of ischemic heart disease, for example, revascularization of ischemic tissue can be encouraged through inhibition of key micro RNAs involved in this process (miR-92a, miR-27b) using antagomirs.^22,23^ Moreover, other approaches have shown effectiveness in reducing pathological cardiac hypertrophy and ischemic heart disease by targeting genes of CaMKII, a kinase involved in maladaptive cardiac remodeling.^24^ Antisense oligonucleotides (ASOs) have also shown therapeutic potential in inherited cardiac manifestations of muscular dystrophies, such as Duchenne muscular dystrophy (DMD). ASOs designed to induce exon skipping can restore expression of partially functional dystrophin, which may in turn improve cardiac muscle stability and function.^25,26^ Emerging data suggest that targeting cardiac involvement with these exon-skipping ASOs could delay or reduce the severity of cardiomyopathy in DMD patients.^27^

The success of GalNAc modified therapeutic oligonucleotides represents an elegant solution to the delivery problem for liver hepatocytes and demonstrates the potential for identifying other small molecule conjugates to redirect delivery to specific tissues. Recently, tannic acid (TA), a polyphenol isolated from plants, has been shown to be an important molecule in influencing cardiac-tissue-targeting. Comprising multiple hydrogen-bond donors, it is suggested that tannic acid derived molecules have enhanced affinity toward proline-rich proteins like collagen and elastin, as a function of high avidity hydrogen bonding.^28,29^ Generally, an extracellular matrix (ECM) is formed of multiple components, but the cardiac ECM is abundant in collagen,^30^ making it especially relevant for TA-mediated targeting. To this end, researchers have shown that when protein or peptide cargos are formulated into nanoparticles with TA (a process termed TANNylation), a 260% increase in heart accumulation was observed.^31^ In another study, TA-formulated cerium-based nanocatalysts showed effective cardiac accumulation and conferred protection against ischemic damage.^32^ The preferential interactions of TA-based nanomaterials with proline-rich ECM components have been further validated in a study that directly assessed aggregation of TA modified and unmodified lipid nanovesicles with collagen.^33^ Indeed, TA is a promising and proven small molecule candidate for enhancing cardiac delivery of therapeutic cargo. However, although TA has demonstrated significant promise for cardiac delivery, most TA-based targeting systems are nanoparticles with undefined ligand densities, and the effect of conjugating defined, stoichiometric numbers of TA moieties to therapeutic cargos remains largely unexplored. Furthermore, for therapeutic oligonucleotides to be effective, both tissue-specific targeting and efficient cellular uptake are essential. Therefore, even if TA enhances cardiac tissue localization, it does not necessarily ensure cellular internalization and subsequent biological activity.

In this study, we aimed to leverage the heart-targeting properties of TA by chemically conjugating the molecule to the passenger (sense) strand of an siRNA duplex. Since it remains unclear whether a single TA molecule is sufficient to confer cardiac targeting, we synthesized and evaluated a series of siRNA conjugates containing up to three TA units. In addition to these TA-based conjugates, we investigated potential synergistic effects by coconjugating TA with other ligands – such as palmitic acid (C16) and AlbuTag (N^6^-4-(4-iodophenyl)­butanoyl-lysine) – which have previously been shown to enhance extrahepatic delivery^34^ and circulation time,^35^ respectively. All conjugates were tested *in vivo* using a model siRNA sequence to enable a robust knockdown readout. Although the TA modifications did not yield the expected enhancement in cardiac gene knockdown, we do observe improved knockdown in the liver compared to naked siRNA. Moreover, these findings offer valuable insights into siRNA conjugate design and establish a synthetic framework for developing polyphenol-containing oligonucleotide conjugates.

## Results and Discussion

### Synthesis of TA-Modified siRNA

To enable precise conjugation of oligonucleotides with TA, a method to modify TA with a single azide group was developed (Figure 1a). The chemical structures of polyphenols, such as TA, present challenges for controlling the distribution of reaction products due to the multiplicity and reactivity of their hydroxyl groups. Moreover, TA contains many hydrolyzable ester bonds that are readily cleaved in acidic and basic conditions or at elevated temperatures to release gallic acid units. These characteristics therefore demand stoichiometrically controlled reactions performed under mild conditions. Briefly, 1 equiv of TA, dissolved in dry DMF, was activated using 1.2 equiv of carbonyldiimidazole (CDI) with stirring at room temperature for 20 min. To this solution, 1.2 equiv of 6-azido hexylamine (in DMF) was added dropwise. The reaction was stirred for 2 h and then quenched with water. Liquid chromatography mass-spectrometry (LC-MS) analysis of this reaction reveals a mixture of TA compounds containing one (TA-N_3_) or two (TA-(N_3_)_2_) azide groups, as well as some unreacted starting material (Figures 1b-d). To interpret the mass spectrometry (MS) data, it is important to note that TA is a polydisperse oligomer of gallic acid units surrounding a central glucose molecule. This results in relatively complex MS spectra of TA derived compounds where multiple peaks arise from molecules containing different numbers of gallic acid units (Figure S1). Importantly, the distribution of peaks that represents unmodified TA molecules shifts upon modification with 6-azido hexylamine (Figure S2). Analytically, we observe the expected mass shift of 168 and 336 mass units for TA-N_3_ and TA-(N_3_)_2_, respectively (Figure 1c). After RP-HPLC and freeze-drying, TA-N_3_ was isolated as a white powder (Figure 1e). Importantly, buffer free HPLC solvents were used to avoid hydrolytic degradation upon freeze-drying.

**Figure 1:**
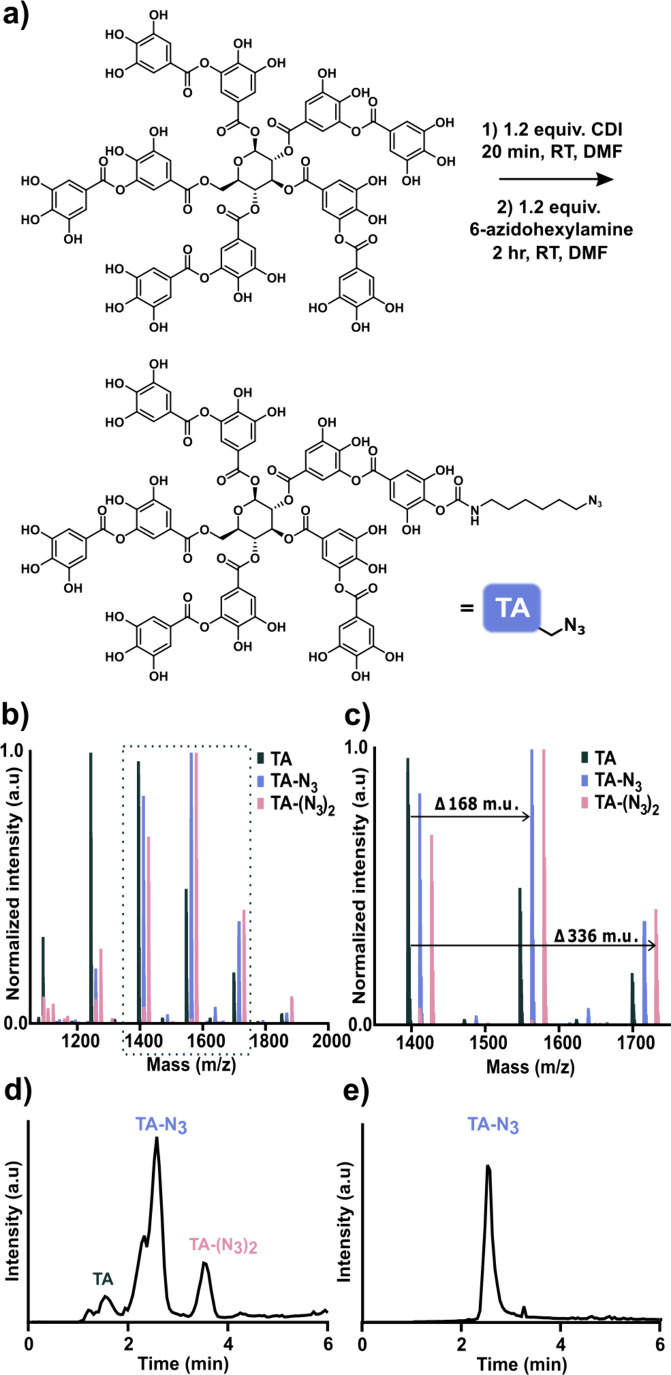
Synthesis and characterization of azide modified TA. (a) Scheme describing conditions for controlled activation and modification of TA to favor mono azide products. One of the many possible mono azide stereomers shown for simplicity. (b) Mass spectrum showing peaks for TA, TA-N_3_ and TA-(N_3_)_2_. Dotted line panel indicates mass region of interest, shown in (c), displaying peak shifts upon addition of one and two azide molecules. (d) Chromatogram of crude reaction products. (e) Chromatogram of isolated TA-N_3_ post HPLC purification.

siRNA conjugates containing either 1, 2, or 3 TA units were synthesized using strain-promoted azide alkyne click (SPAAC) chemistry. Briefly, the termini of the sense strand were modified with 1, 2, or 3 bicyclononyn (BCN) groups and then reacted with TA-N_3_ (TA containing a single azide modification) to yield products with a defined number of TA units (Scheme 1). For mono and bis conjugates, TA-N_3_ units were conjugated to either 5′ or 5′ and 3′ termini of the sense strand, respectively (Scheme 1a,b, Supporting Information 4.1 and 4.2). However, to synthesize a structure containing 3 TA units, a branching linker was first introduced at the 3′ position (Scheme 1c) using *N*-hydroxysuccinimide (NHS) chemsitry (Supporting Information 4.3). Final conjugates were isolated using HPLC, desalted and freeze-dried prior to annealing with antisense RNA for use *in vivo*. LCMS analysis confirmed the identity and purity of each conjugate (Supporting Information 4). We chose to synthesize all conjugates using a model siRNA sequence that targets murine superoxide dismutase (Sod1) gene as validated sequences are available and it is widely expressed throughout the body.^36^ The full sequence as well as backbone and termini modifications are described in Scheme S1. To ensure that TA modifications did not induce unwanted aggregation of siRNA molecules, we performed dynamic light scattering (DLS) experiments. These measurements confirmed the hydrodynamic radii (5–10 nm) expected for monomeric siRNA conjugates (Figure S12).

**Scheme 1:**
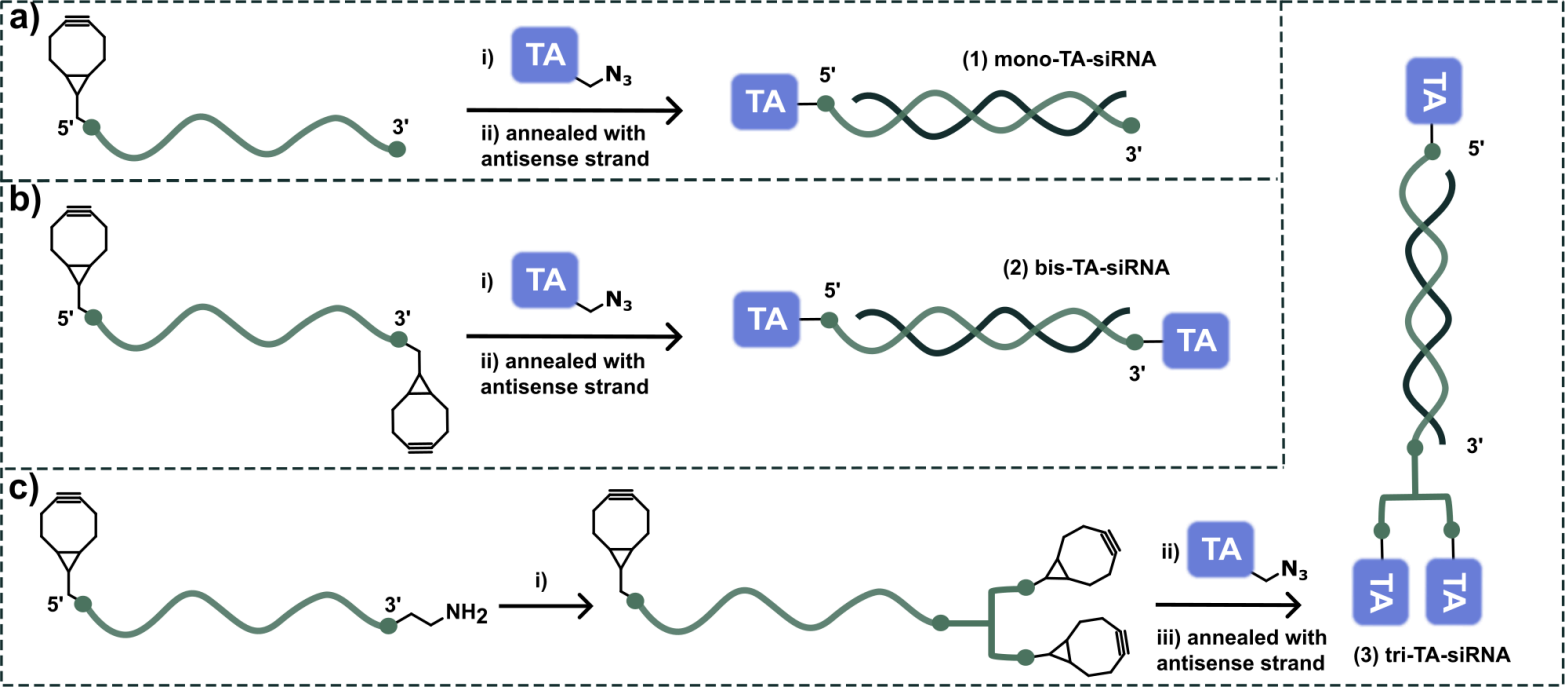
Overview of the Key Steps to Generate a Series of siRNA Conjugates Containing 1-3 TA Units^a^

a (a) (i) A single TA unit is installed via a single BCN group at the 5′ position of the sense strand followed by (ii) subsequent annealing with antisense oligo at 90°C and slow cooling from 90-37°C over 45 min. (b) (i) Bis TA conjugation of sense strand via BCN groups at both the 5′ and 3′ termini of the sense strand followed by (ii) annealing protocol to antisense. (c) (i) Two BCN groups are first introduced to the 3′ end of the sense strand using NHS chemistry to generate a structure containing 3 BCN groups. (ii) TA conjugation to all 3 BCN sites via click reactions followed by (iii) annealing protocol to antisense.

### Evaluating Efficacy of TA-siRNA Conjugates *In Vivo*

We hypothesized that increasing the number of TA units per siRNA duplex would lead to greater activity of siRNA to the heart, mediated by enhanced cardiac tissue avidity from multiple TA units. First, to confirm that these conjugates were still active, we performed *in vitro* analysis. Sod1 mRNA levels measured in transfected neuro-2a cells showed similar knockdown across all conjugates to unconjugated (naked) Sod1 siRNA (Figure S13). However, *in vitro* efficacy of siRNA conjugates provides minimal predictive understanding of organ specific activity. Therefore, to test our hypothesis, we injected each conjugate or an unmodified (naked) siRNA control intravenously (IV) in mice (female NMRI mice, n = 4–5 per group, 600 nmols/kg, corresponding to approximately 9.1–12.9 mg/kg of siRNA with respect to the molecular weight of the conjugate) and harvested organs at day 7 post injection for analysis by RT-qPCR (Supporting Information 7). This dosage is in line with reported IV siRNA treatments,^37,38^ and our unpublished data utilizing this sequence suggests that this dose provides sufficient potency to observe differences between conjugation chemistries. Whole tissues were homogenized during the RNA isolation protocol to ensure homogeneous sampling across the tissue. Using qPCR, Sod1 expression was analyzed relative to the housekeeping gene Gapdh, and normalized to the PBS-treated control group (Figure 2, Supporting Information 7.1). Interestingly, when analyzing the liver, we observe significant knockdown for all TA conjugates compared to naked siRNA, with bis-TA (2) eliciting just over 40% knockdown, suggesting that these modifications influence accumulation of siRNA in cells of the liver. Moreover, there seems to be minimal differences in knockdown between siRNAs modified with 1, 2, and 3 units of TA. Surprisingly, however, TA conjugates do not significantly improve knockdown in the heart, compared to naked siRNA. While these data go against our hypothesis, it is an important observation in the context of the literature regarding TA mediated drug delivery. These findings suggest that the modification of siRNA with low numbers of TA units is insufficient to enhance siRNA activity in the heart and that, perhaps, heart targeting is an emergent property of structures comprising greater numbers of TA ligands.

**Figure 2:**
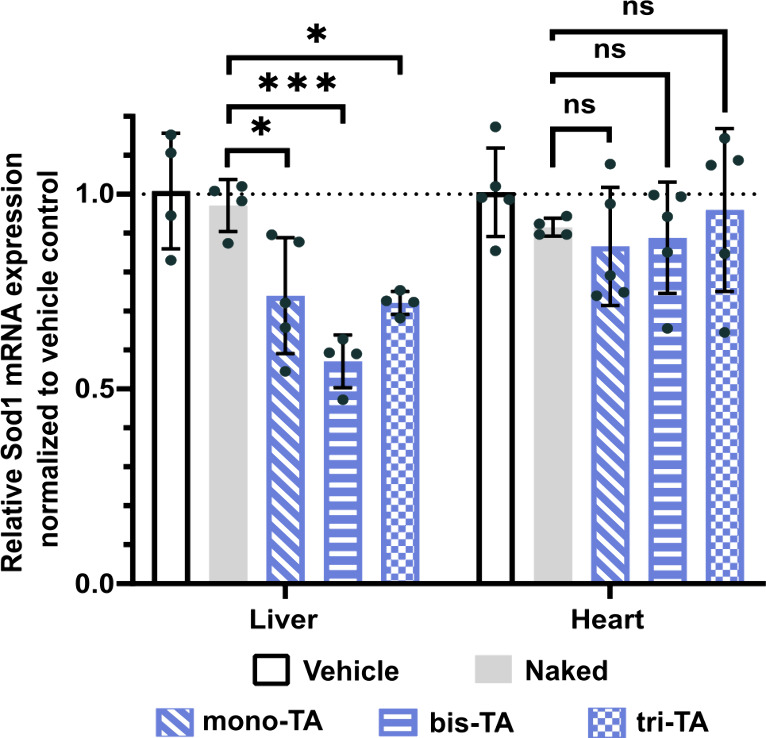
Sod1 knockdown in liver and heart tissue normalized to vehicle control for TA-siRNA conjugates 7 days post IV injection (600 nmols/kg, *n* = 4–5). Statistical analysis was performed using ordinary one-way ANOVA followed by Dunnett’s multiple comparisons test to compare groups against the naked siRNA group for each tissue separately.

The increase in knockdown observed in the liver for TA conjugates, compared to naked siRNA is an interesting and unexpected result. While this observation might suggest that TA modifications enhance the accumulation and/or cellular uptake in this tissue, it could also be explained by myriad potential mechanisms including increased metabolic stability; improved pharmacodynamic efficacy or enhanced endosomal escape kinetics. Further investigations are required to fully understand the mechanism driving this effect. It is well established that therapeutic siRNAs, are rapidly cleared from the blood (within minutes) since they are below the molecular weight cut off of glomerular filtration in the kidneys (40–50 kDa),^39^ resulting in a short temporal window for cellular uptake into organs not involved in clearance.^40^ Furthermore, initial accumulation in the liver is also characteristic of such biomolecules, so perhaps this is why the effect of TA modifications are most prominent in the liver. We hypothesized that in order to observe extrahepatic activity of these TA modified conjugates, it may be important to enhance the circulation lifetime to promote accumulation and cellular uptake into other tissues. One way to do this is by modifying siRNA with molecules that associate with components of the blood to extend the lifetime of these molecules in circulation.^41,42^ We postulated that it may be possible to observe the delivery effects of TA modification in extrahepatic organs when combined in siRNA conjugates that are designed to persist in blood. To investigate this, we designed and synthesized hetero-bifunctional conjugates comprising both a TA modification and an albumin binding motif (Figure 3a), at the 5′ and 3′ termini, respectively. We compared the knockdown efficiency of these hetero-bifunctional conjugates to those possessing only the 3′ albumin binding motifs. The albumin binding motifs include a C16 lipid (palmitate) or a small molecule N^6^-4-(4-iodophenyl)­butanoyl-lysine, AlbuTag, both of which have been previously studied for their albumin binding properties.^35^

**Figure 3:**
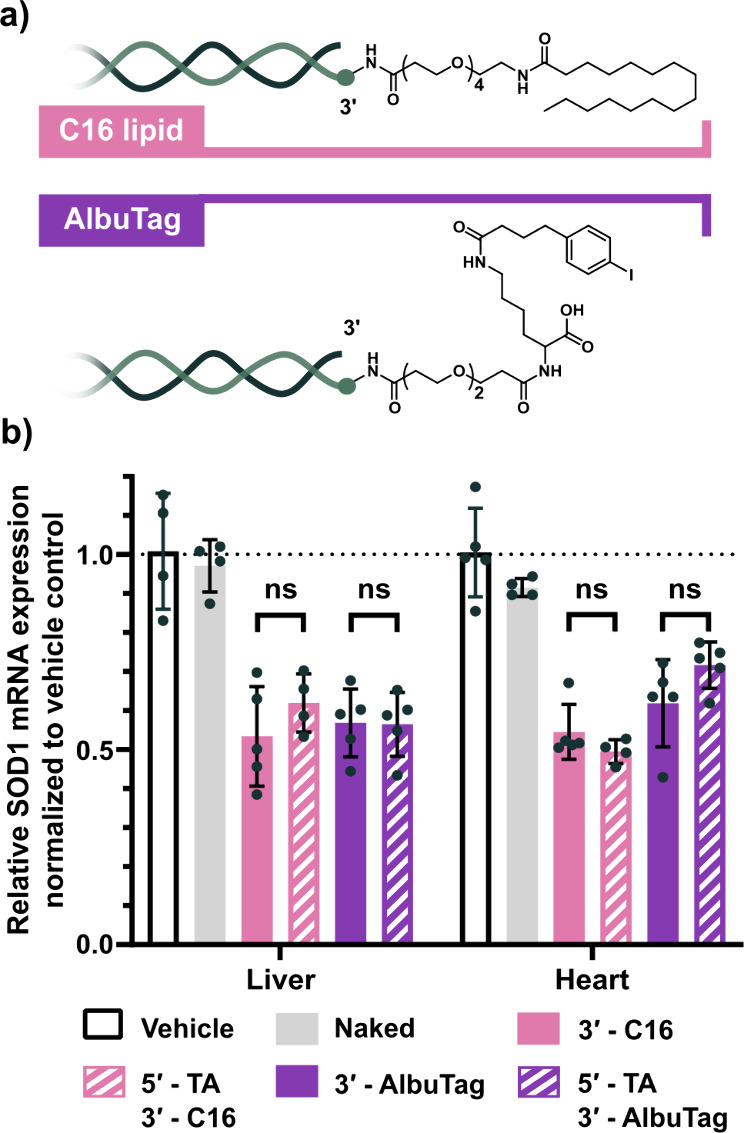
Investigating synergistic effects of hetero-bifunctional siRNA conjugates on tissue specific knockdown. (a) Chemical structures of albumin binding modifications at 3′ terminus of sense strand. (b) Sod1 knockdown in liver and heart tissue normalized to vehicle control for TA-siRNA conjugates 7 days post IV injection (600 nmols/kg, *n* = 4–5). Statistical analysis was performed using ordinary one-way ANOVA followed by Dunnett’s multiple comparisons test to compare treatment groups.

*In vivo* experiments were performed for the albumin binding conjugates, following the same protocol as described in Supporting Information 7. For all conjugates containing either C16 or AlbuTag, we observed significant Sod1 mRNA knockdown in both the liver and the heart. Indeed, this was an expected result for the C16 conjugates, as such conjugates have been previously been shown to enhance activity of an ASO in heart and quadriceps.^34^ For all conjugates, similar knockdown (between 39 and 47%) is observed in the liver (Figure 3b). In the heart, slightly greater knockdown is achieved by C16 conjugates (45 and 51%) compared to AlbuTag conjugates (38 and 28%). These results clearly reveal the impact of incorporating albumin binding motifs into the design of siRNA conjugates on their hepatic and extrahepatic activity and provide insight into how two very structurally different small-molecule albumin binders compare. Importantly, there is no significant difference in knockdown when we compare the hetero-bifunctional C16 or AlbuTag (containing both TA at 5′ and a 3′ albumin binding motif) with their monofunctional counterparts (only 3′ albumin binding motif) in either the liver or the heart. Therefore, under these conditions, we can conclude that incorporating a single TA unit into albumin binding conjugates does not greatly influence their functional delivery and knockdown efficiencies. In fact, these results further confirm that a single TA modification is not sufficient to confer the heart-targeting properties that are reported for structures containing many copies of the molecule.

## Conclusions

In summary, we developed a synthetic strategy for introducing a single azide functionality into tannic acid, enabling the synthesis of structurally defined siRNA–TA conjugates with one to three TA units. While these conjugates displayed enhanced knockdown in the liver relative to naked siRNA, they did not significantly improve knockdown in the heart, indicating that a small number of TA ligands is insufficient to drive functional cardiac activity. By contrast, conjugation with albumin-binding motifs, including both a C16 lipid and the small-molecule binder AlbuTag, conferred robust knockdown in both hepatic and extrahepatic tissues, underscoring the importance of circulation half-life extension for extrahepatic targeting via improved pharmacokinetics. Notably, the addition of a single TA unit to albumin-binding conjugates did not further improve their efficacy, suggesting that TA-mediated delivery effects may only emerge in the context of higher-valency or multimeric, larger architectures.

While this work focused on the synthesis and study of molecularly defined TA-siRNA conjugates, we believe these conjugation strategies could also inform the development of nanoparticle delivery-based systems. For example, the formulation of siRNA-TA nanoparticles, prepared by cross-linking of TA ligands,^43,44^ may have structures and properties analogous to spherical nucleic acids.^45,46^ Furthermore, the pH labile nature of the ester bonds in TA could also impart stimuli responsive degradation and release of siRNA intracellularly. Together, the synthesis of structurally defined TA conjugates and their *in vivo* analysis provide mechanistic insight into the roles of TA and albumin-binding motifs in conjugated siRNA activity *in vivo* and highlight key design considerations for future conjugates and nanomaterials aimed at achieving tissue-selective delivery beyond the liver. For example, these findings indicate the need for higher valency TA architectures to be explored in future work in order to enable effective siRNA activity in the heart.

Safety and Hazard Statement. No unexpected or unusually high safety hazards were encountered.

## Supplementary Material



## Notes

Oligonucleotide structures, sequences and molecular weights; Detailed synthesis and characterization of TA-N_3_; Synthesis methods for all conjugates; Mass spectrometry data for all conjugates; Dynamic Light Scattering (DLS); *In vitro* evaluation of conjugates; Further details regarding animal experiments (PDF)

## References

[1] Hall J. (2023). Future Directions
for Medicinal Chemistry in the Field of Oligonucleotide Therapeutics. RNA.

[2] Naeem S., Zhang J., Zhang Y., Wang Y. (2025). Nucleic Acid Therapeutics:
Past, Present, and Future. Molecular Therapy
Nucleic Acids.

[3] Smith C. I. E., Zain R. (2019). Therapeutic Oligonucleotides: State of the Art. Annual Review of Pharmacology and Toxicology.

[4] Egli M., Manoharan M. (2023). Chemistry,
Structure and Function of Approved Oligonucleotide
Therapeutics. Nucleic Acids Res..

[5] Springer A.
D., Dowdy S. F. (2018). GalNAc-siRNA
Conjugates: Leading the Way for Delivery
of RNAi Therapeutics. Nucleic Acid Therapeutics.

[6] Roberts T. C., Langer R., Wood M. J. A. (2020). Advances in Oligonucleotide Drug
Delivery. Nat. Rev. Drug Discov.

[7] Simonsen J. B. (2024). Lipid Nanoparticle-Based
Strategies for Extrahepatic Delivery of Nucleic Acid Therapies - Challenges
and Opportunities. J. Controlled Release.

[8] Chen L., Hong W., Ren W., Xu T., Qian Z., He Z. (2021). Recent Progress in Targeted Delivery
Vectors Based on Biomimetic
Nanoparticles. Sig Transduct Target Ther.

[9] Cheng M. H. Y., Zhang Y., Fox K., Leung J., Strong C., Kang E., Chen Y., Tong M., Bommadevara H., Jan E., Ip O. Y. L., Rodríguez-Rodríguez C., Saatchi K., Häfeli U. O., Abdolahzadeh A., Witzigmann D., Cullis P. R. (2025). Liposomal Lipid
Nanoparticles for
Extrahepatic Delivery of mRNA. Nat. Commun..

[10] Pal S., Singla D., Canete R. C., Darkwah J. B., Cannata J. N., Hunte M. L., Penales I. B., Szczepanek S. M., Ducongé F., Smilowitz H. M., Rouge J. L. (2025). Virus-Inspired mRNA
Delivery Vehicle Enabled by a Multilayered Nucleic Acid Nanocapsule. ACS Nano.

[11] Pal S., Cannata J. N., Rouge J. L. (2025). Nucleic
Acid Nanocapsules as a New
Platform to Deliver Therapeutic Nucleic Acids for Gene Regulation. Acc. Chem. Res..

[12] Mangla P., Vicentini Q., Biscans A. (2023). Therapeutic Oligonucleotides: An
Outlook on Chemical Strategies to Improve Endosomal Trafficking. Cells.

[13] Biscans A., Coles A., Haraszti R., Echeverria D., Hassler M., Osborn M., Khvorova A. (2019). Diverse Lipid Conjugates
for Functional Extra-Hepatic siRNA Delivery in Vivo. Nucleic Acids Res..

[14] Biscans A., Ly S., McHugh N., Cooper D. A., Khvorova A. (2022). Engineered Ionizable
Lipid siRNA Conjugates Enhance Endosomal Escape but Induce Toxicity
in Vivo. J. Controlled Release.

[15] Ernsting M. J., Murakami M., Roy A., Li S.-D. (2013). Factors Controlling
the Pharmacokinetics, Biodistribution and Intratumoral Penetration
of Nanoparticles. J. Controlled Release.

[16] Hammond S. M., Aartsma-Rus A., Alves S., Borgos S. E., Buijsen R. A. M., Collin R. W. J., Covello G., Denti M. A., Desviat L. R., Echevarría L., Foged C., Gaina G., Garanto A., Goyenvalle A. T., Guzowska M., Holodnuka I., Jones D. R., Krause S., Lehto T., Montolio M., Van Roon-Mom W., Arechavala-Gomeza V. (2021). Delivery of Oligonucleotide-based
Therapeutics: Challenges and Opportunities. EMBO Molecular Medicine.

[17] Kalita T., Dezfouli S. A., Pandey L. M., Uludag H. (2022). siRNA Functionalized
Lipid Nanoparticles (LNPs) in Management of Diseases. Pharmaceutics.

[18] Biscans A., Caiazzi J., McHugh N., Hariharan V., Muhuri M., Khvorova A. (2021). Docosanoic Acid Conjugation to siRNA
Enables Functional and Safe Delivery to Skeletal and Cardiac Muscles. Molecular Therapy.

[19] Matsuda S., Keiser K., Nair J. K., Charisse K., Manoharan R. M., Kretschmer P., Peng C. G. V., V Kel’in A., Kandasamy P., Willoughby J. L. S., Liebow A., Querbes W., Yucius K., Nguyen T., Milstein S., Maier M. A., Rajeev K. G., Manoharan M. (2015). siRNA Conjugates Carrying Sequentially
Assembled Trivalent N-Acetylgalactosamine Linked Through Nucleosides
Elicit Robust Gene Silencing In Vivo in Hepatocytes. ACS Chem. Biol..

[20] Lucas T., Bonauer A., Dimmeler S. (2018). RNA Therapeutics
in Cardiovascular
Disease. Circ. Res..

[21] Mainkar G., Ghiringhelli M., Zangi L. (2025). The Potential of RNA Therapeutics
in Treating Cardiovascular Disease. Drugs.

[22] Bonauer A., Carmona G., Iwasaki M., Mione M., Koyanagi M., Fischer A., Burchfield J., Fox H., Doebele C., Ohtani K., Chavakis E., Potente M., Tjwa M., Urbich C., Zeiher A. M., Dimmeler S. (2009). MicroRNA-92a Controls
Angiogenesis and Functional Recovery of Ischemic Tissues in Mice. Science.

[23] Veliceasa D., Biyashev D., Qin G., Misener S., Mackie A. R., Kishore R., Volpert O. V. (2015). Therapeutic
Manipulation of Angiogenesis
with miR-27b. Vascular Cell.

[24] Yao Y., Li F., Zhang M., Jin L., Xie P., Liu D., Zhang J., Hu X., Lv F., Shang H., Zheng W., Sun X., Duanmu J., Wu F., Lan F., Xiao R.-P., Zhang Y. (2022). Targeting CaMKII-Δ9
Ameliorates
Cardiac Ischemia/Reperfusion Injury by Inhibiting Myocardial Inflammation. Circ. Res..

[25] Echigoya Y., Nakamura A., Nagata T., Urasawa N., Lim K. R. Q., Trieu N., Panesar D., Kuraoka M., Moulton H. M., Saito T., Aoki Y., Iversen P., Sazani P., Kole R., Maruyama R., Partridge T., Takeda S., Yokota T. (2017). Effects of Systemic Multiexon Skipping
with Peptide-Conjugated Morpholinos in the Heart of a Dog Model of
Duchenne Muscular Dystrophy. Proc. Natl. Acad.
Sci. U. S. A..

[26] Etxaniz U., Marks I., Albin T., Diaz M., Bhardwaj R., Anderson A., Tyaglo O., Hoang T., Missinato M. A., Svensson K., Badillo B., Kovach P. R., Leung L., Cochran M., Kwon H. W., Ahad Shah M. N., Maruyama R., Yokota T., Doppalapudi V. R., Darimont B., Younis H. S., Flanagan W. M., Levin A. A., Huang H., Karamanlidis G. (2025). AOC 1044 Induces Exon 44 Skipping
and Restores Dystrophin Protein in Preclinical Models of Duchenne
Muscular Dystrophy. Nucleic Acids Res..

[27] Shiba N., Yang X., Sato M., Kadota S., Suzuki Y., Agata M., Nagamine K., Izumi M., Honda Y., Koganehira T., Kobayashi H., Ichimura H., Chuma S., Nakai J., Tohyama S., Fukuda K., Miyazaki D., Nakamura A., Shiba Y. (2023). Efficacy of Exon-Skipping Therapy
for DMD Cardiomyopathy with Mutations in Actin Binding Domain 1. Molecular Therapy Nucleic Acids.

[28] Sarker P., Jani P. K., Hsiao L. C., Rojas O. J., Khan S. A. (2023). Interacting
Collagen and Tannic Acid Particles: Uncovering pH-Dependent Rheological
and Thermodynamic Behaviors. J. Colloid Interface
Sci..

[29] Velmurugan P., Singam E. R. A., Jonnalagadda R. R., Subramanian V. (2014). Investigation
on Interaction of Tannic Acid with Type I Collagen and Its Effect
on Thermal, Enzymatic, and Conformational Stability for Tissue Engineering
Applications. Biopolymers.

[30] Karamanos N. K., Theocharis A. D., Piperigkou Z., Manou D., Passi A., Skandalis S. S., Vynios D. H., Orian-Rousseau V., Ricard-Blum S., Schmelzer C. E. H., Duca L., Durbeej M., Afratis N. A., Troeberg L., Franchi M., Masola V., Onisto M. (2021). A Guide to
the Composition and Functions of the Extracellular
Matrix. FEBS Journal.

[31] Shin M., Lee H.-A., Lee M., Shin Y., Song J.-J., Kang S.-W., Nam D.-H., Jeon E. J., Cho M., Do M., Park S., Lee M. S., Jang J.-H., Cho S.-W., Kim K.-S., Lee H. (2018). Targeting Protein and Peptide Therapeutics
to the Heart via Tannic Acid Modification. Nat.
Biomed Eng..

[32] Wang L., Qiu S., Li X., Zhang Y., Huo M., Shi J. (2023). Myocardial-Targeting
Tannic Cerium Nanocatalyst Attenuates Ischemia/Reperfusion Injury. Angew. Chem., Int. Ed..

[33] Jeon M., Ryu J. S., Kim S. E., Seo J. Y., Cho H. D., Kim S., Lee S., Kim S., Kim J. W. (2024). Selective Binding
of Tannic Acid-Conjugated Lipid Nanovesicles to Proline-Rich Proteins
Enhances Transdermal Lipophilic-Antioxidant Delivery. ACS Appl. Bio Mater..

[34] Prakash T. P., Mullick A. E., Lee R. G., Yu J., Yeh S. T., Low A., Chappell A. E., Østergaard M. E., Murray S., Gaus H. J., Swayze E. E., Seth P. P. (2019). Fatty Acid
Conjugation Enhances Potency
of Antisense Oligonucleotides in Muscle. Nucleic
Acids Res..

[35] Zorzi A., Linciano S., Angelini A. (2019). Non-Covalent Albumin-Binding Ligands
for Extending the Circulating Half-Life of Small Biotherapeutics. Medchemcomm.

[36] Brown K. M., Nair J. K., Janas M. M., Anglero-Rodriguez Y. I., Dang L. T. H., Peng H., Theile C. S., Castellanos-Rizaldos E., Brown C., Foster D., Kurz J., Allen J., Maganti R., Li J., Matsuda S., Stricos M., Chickering T., Jung M., Wassarman K., Rollins J., Woods L., Kelin A., Guenther D. C., Mobley M. W., Petrulis J., McDougall R., Racie T., Bombardier J., Cha D., Agarwal S., Johnson L., Jiang Y., Lentini S., Gilbert J., Nguyen T., Chigas S., LeBlanc S., Poreci U., Kasper A., Rogers A. B., Chong S., Davis W., Sutherland J. E., Castoreno A., Milstein S., Schlegel M. K., Zlatev I., Charisse K., Keating M., Manoharan M., Fitzgerald K., Wu J.-T., Maier M. A., Jadhav V. (2022). Expanding
RNAi Therapeutics to Extrahepatic Tissues with Lipophilic Conjugates. Nat. Biotechnol..

[37] Nair J. K., Attarwala H., Sehgal A., Wang Q., Aluri K., Zhang X., Gao M., Liu J., Indrakanti R., Schofield S., Kretschmer P., Brown C. R., Gupta S., Willoughby J. L. S., Boshar J. A., Jadhav V., Charisse K., Zimmermann T., Fitzgerald K., Manoharan M., Rajeev K. G., Akinc A., Hutabarat R., Maier M. A. (2017). Impact of Enhanced Metabolic Stability
on Pharmacokinetics
and Pharmacodynamics of GalNAc-siRNA Conjugates. Nucleic Acids Res..

[38] Fakih H. H., Tang Q., Summers A., Gross K. Y., Rachid M. O., Okamura K., Martinez N., Sleiman H. F., Harris J. E., Khvorova A. (2025). Albumin-Binding Dendritic
siRNA Improves Delivery and
Efficacy to Solid Tumors in a Melanoma Model. Molecular Therapy Nucleic Acids.

[39] Roksnoer L. C. W., Heijnen B. F. J., Nakano D., Peti-Peterdi J., Walsh S. B., Garrelds I. M., van Gool J. M. G., Zietse R., Struijker-Boudier H.
A. J., Hoorn E. J., Danser A. H. J. (2016). On the
Origin of Urinary Renin. Hypertension.

[40] Iversen F., Yang C., Dagnæs-Hansen F., Schaffert D. H., Kjems J., Gao S. (2013). Optimized siRNA-PEG Conjugates for
Extended Blood Circulation and Reduced Urine Excretion in Mice. Theranostics.

[41] Godinho B. M. D. C., Knox E. G., Hildebrand S., Gilbert J. W., Echeverria D., Kennedy Z., Haraszti R. A., Ferguson C. M., Coles A. H., Biscans A., Caiazzi J., Alterman J. F., Hassler M. R., Khvorova A. (2022). PK-Modifying Anchors Significantly Alter Clearance
Kinetics, Tissue Distribution, and Efficacy of Therapeutics siRNAs. Mol. Ther Nucleic Acids.

[42] Osborn M. F., Coles A. H., Biscans A., Haraszti R. A., Roux L., Davis S., Ly S., Echeverria D., Hassler M. R., Godinho B. M. D. C., Nikan M., Khvorova A. (2019). Hydrophobicity
Drives the Systemic Distribution of Lipid-Conjugated siRNAs via Lipid
Transport Pathways. Nucleic Acids Res..

[43] Sahiner N. (2021). Self-Crosslinked
Ellipsoidal Poly­(Tannic Acid) Particles for Bio-Medical Applications. Molecules.

[44] Sahiner N., Sagbas S., Aktas N. (2015). Single Step Natural
Poly­(Tannic Acid)
Particle Preparation as Multitalented Biomaterial. Materials Science and Engineering: C.

[45] Banga R. J., Meckes B., Narayan S. P., Sprangers A. J., Nguyen S. T., Mirkin C. A. (2017). Cross-Linked Micellar
Spherical Nucleic
Acids from Thermoresponsive Templates. J. Am.
Chem. Soc..

[46] Forsyth C. M., Chan R. R., Fink T. D., Kang J., Cohen J. D., Petrosko S. H., Mirkin C. A. (2026). Spherical Nucleic Acids: Turning
Synthetic Advances and Fundamental Discovery into Translational Breakthroughs
in Chemistry, Materials Development, Biology, and Medicine. Acc. Chem. Res..

