# Supplementary material for: Investigating the In Vivo Performance of Tannic Acid-Modified siRNA in the Heart and Liver
Source: Bioconjug Chem. 2026 May 27;37(6):1121–8. doi: 10.1021/acs.bioconjchem.6c00122 (PMC13281385; doi:10.1021/acs.bioconjchem.6c00122)
Supplement: Supplementary file 1 [file bc6c00122_si_001.pdf]

Supporting Information for
Investigating the *in vivo* performance of tannic acid modified siRNA in the heart and liver

O. G. Hayes †‡, J. Rädler †‡, E. Filipiak †‡, T. Czapik †‡, Y. Huang †‡, M. Ojansivu †‡§, O. Saher †‡, M. Bood‡, R. Zain †‡§⊥, S. EL. Andaloussi †‡§* and M. Honcharenko †‡*

†Division of Biomolecular and Cellular Medicine, Department of Laboratory Medicine, Karolinska Institutet, Huddinge, 14152, Stockholm, Sweden.

‡Department of Cellular Therapy and Allogeneic Stem Cell Transplantation (CAST), Karolinska University Hospital, 14186 Stockholm, Sweden.

§Karolinska ATMP (Advanced Therapy Medicinal Products) Center, Karolinska Institutet, 14152 Stockholm, Sweden

⊥Center for Rare Diseases, Clinical Genetics and Genomics, Karolinska University Hospital, SE-17176, Stockholm, Sweden

‡Nucleic Acid Therapeutics, Oligonucleotides and Targeted Delivery, Discovery Sciences, AstraZeneca, 43153, Mölndal, Sweden

Table of Contents

1. List of abbreviations 3
2. Structure of oligonucleotides 3
 2.1 General structure of chemically modified Sod1 siRNA 3
 2.2 Table of oligonucleotide sequences and molecular weights..4
3. Synthesis and characterization of azide modified tannic-acid (TA-N_3) 4
4. Synthesis of TA-modifed siRNA conjugates 6
 4.1 mono-TA-siRNA 6
 4.2 bis-TA-siRNA 6
 4.3 tri-TA-siRNA..7
 4.4 siRNA-peg4-C16 9
 4.5 TA-siRNA-peg4-C16 10
 4.6 siRNA-AlbuTag..11
 4.7 TA-siRNA-AlbuTag..13
 4.8 LC-MS method 14
5. DLS of TA conjugates 14
6. In vitro evaluation of siRNA conjugates 15
7. Animal experiments..16
 7.1 In vivo experiments and knockdown analysis by qPCR 16

1. List of abbreviations

ACN - Acetonitrile

AS – Antisense strand

BCN - Bicyclononyne

DMF - Dimethylformamide

DMSO – Dimethyl sulfoxide

GA – Gallic acid

NHS - N-Hydroxysuccinimide

Peg – Polyethylene glycol

PBS – Phosphate buffered saline

SS – Sense strand

TA – Tannic acid

TEAA – Triethyl ammonium acetate

2. Structure of oligonucleotides

2.1 General structure of chemically modified Sod1 siRNA

Oligonucleotides used in this study was designed at AstraZeneca and acquired from Axolabs GmbH. The sequences and backbone chemical modifications are described in scheme S1.

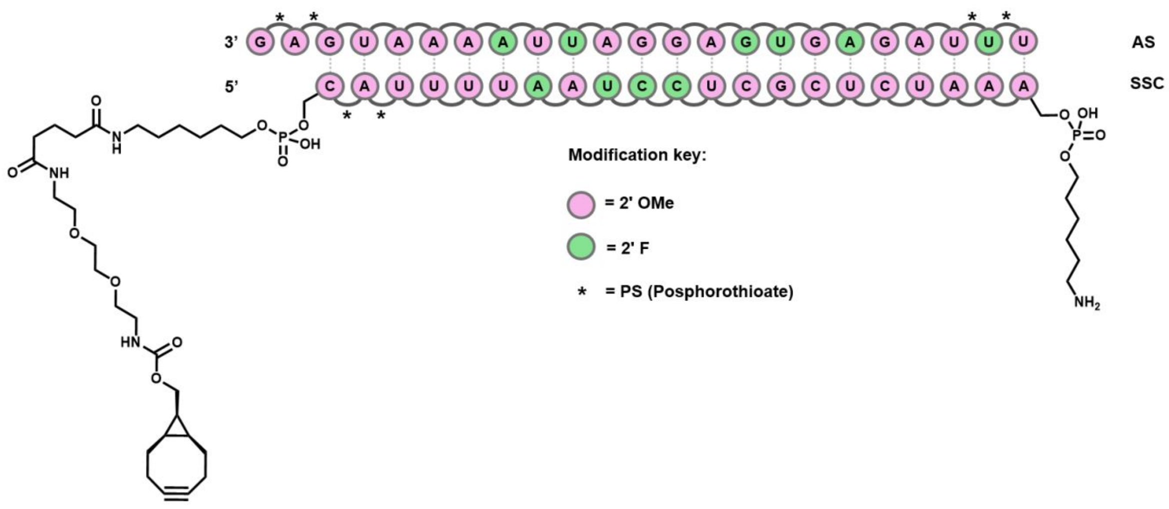

Scheme S1. Schematic representation of Sod1 siRNA structure using comprising antisense (AS) and sense strand B (SSB).

2.2 Table of oligonucleotide sequences and molecular weights

Name	Sequence (5′ to 3′)	Calculated Mw (Da)	Found Mw* (Da)
SSA (Sense Strand A)	BCN-CAUUUUAAUCCUCACUCUAAA	7401.1	7399.9
SSB (Sense Strand B)	BCN-CAUUUUAAUCCUCACUCUAAA-BCN	8000.8	8000.6
SSC (Sense Strand C)	BCN-CAUUUUAAUCCUCACUCUAAA-NH2	7580.3	7579.4
AS (Antisense)	UUUAGAGUGAGGAUUAAAAUGAG	7775.4	7774.4
TA-SSA	TA-CAUUUUAAUCCUCACUCUAAA	9270.3	9270
TA-SSB-TA	TA-CAUUUUAAUCCUCACUCUAAA-TA	11739.2	11740
TA-SSC-bisTA	TA-CAUUUUAAUCCUCACUCUAAA-$(TA)_2$	14220.2	14129
SSC-peg4-C16	CAUUUUAAUCCUCACUCUAAA-peg4-C16	8066.1	8066
TA-SSC-peg4-C16	TA-CAUUUUAAUCCUCACUCUAAA-peg4-C16	9935.3	9935
SSC-AlbuTag	CAUUUUAAUCCUCACUCUAAA-AlbuTag	8168.6	8168
TA-SSC-AlbuTag	TA-CAUUUUAAUCCUCACUCUAAA-AlbuTag	10037.8	9885 (-1 **GA unit**)

*for structures containing TA modifications, deconvoluted mass specra show a distribution masses atrribited to +/- gallic acid units.

GA units = gallic acid unit (Δ 153.12 Da)

3. Synthesis and characterization of azide modified tannic-acid (TA-N_3)

In a typical reaction, 1 equiv. of TA, dissolved in dry DMF, was activated using 1.2 equivalents of carbonyldiimidazole (CDI) with stirring at room temperature for 20 mins. To this solution, 1.2 equivalents of 6-azido hexylamine (in DMF) was added dropwise. The reaction was stirred for 2 hours and then quenched with water. The crude reaction mixture was added to a separatory funnel and extracted with 3x washes of ethylacetate. The aqueous phase was collected and products purified using RF-HPLC and buffer free mobile phases (A= 10% ACN in H_2O, B= 100% ACN). Fractions containing TA-N_3 were collected and freeze dried to give a fluffy, white solid. Typical yield between 40-50%. LC-MS analysis of pure TA-N_3 confirmed the mass: Expected 1869.2 Da, found:1716.3 Da (-1 GA unit).

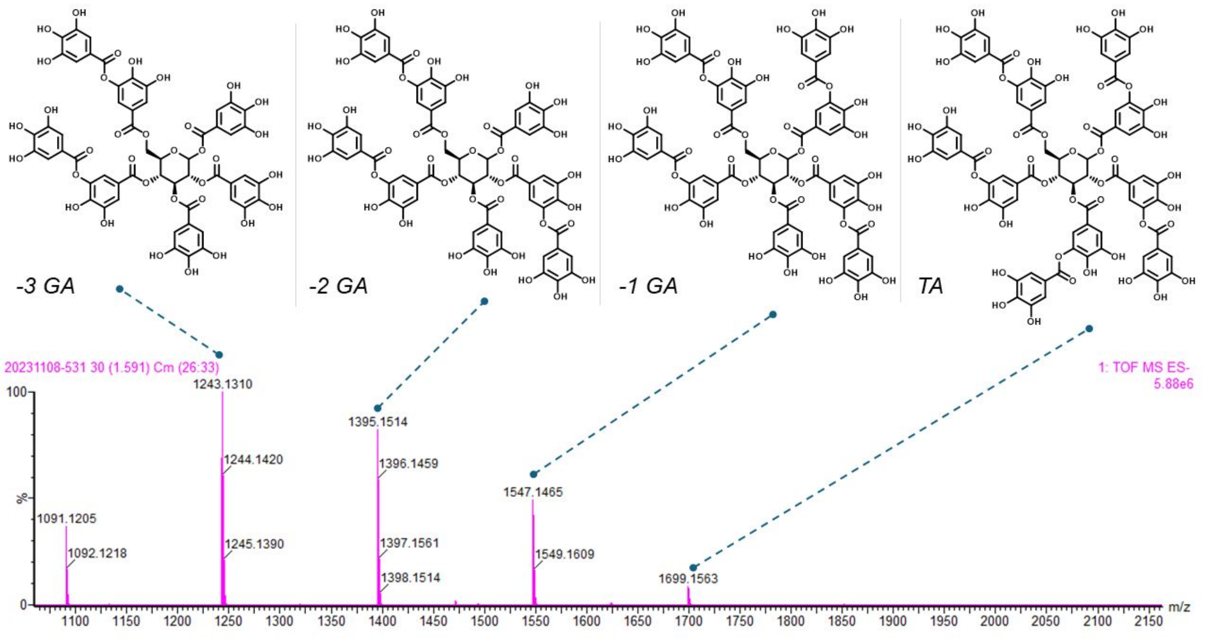

Figure S1. Typical mass spectrum of TA (bottom) with peaks assigned to representative structures of a possible stereomer of each mass (top).

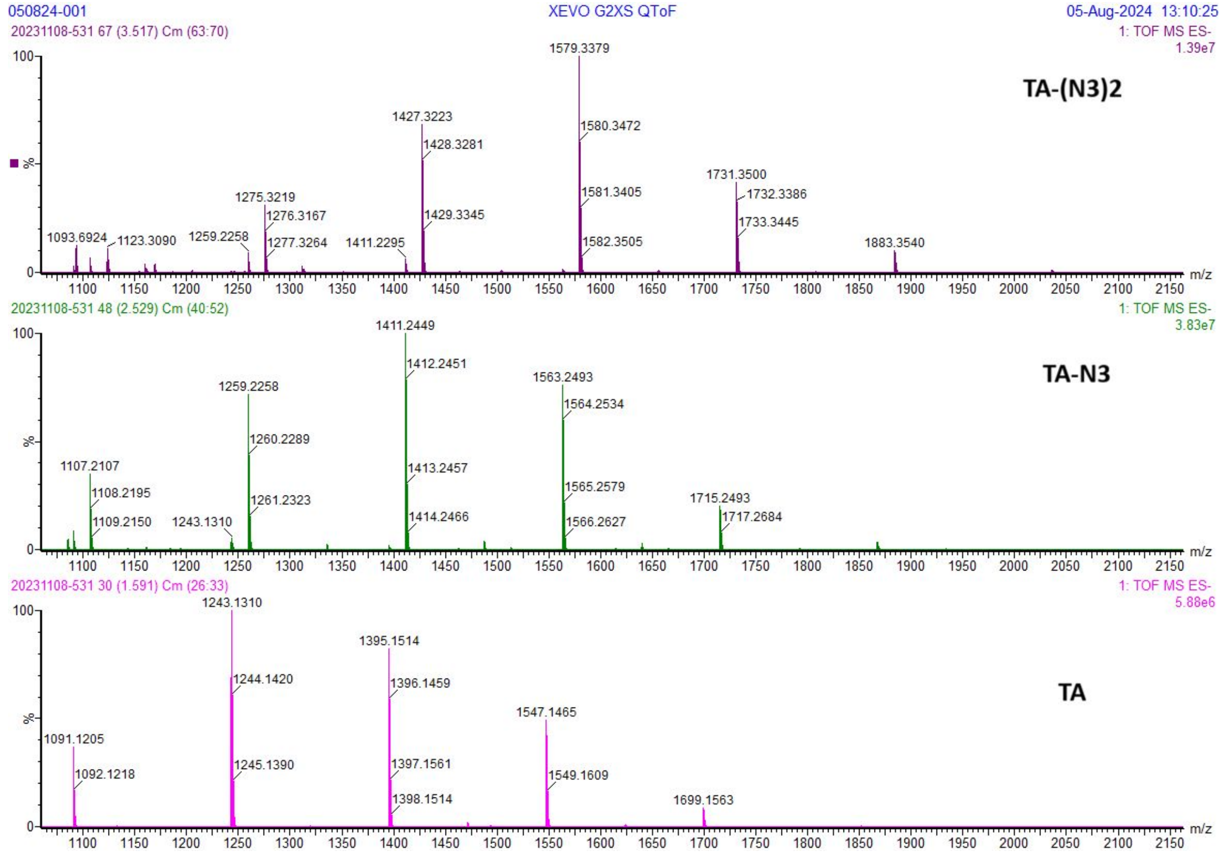

Figure S2. Mass specra of TA-$(N_3)_2$ (top), TA-N_3 (middle), and TA (bottom)

4. Synthesis of TA-modifed siRNA conjugates

4.1 mono-TA-siRNA

In a 2mL eppendorf, 2 equiv. of TA-N_3, dissolved in H_2O, were added to 1 equiv. of SSA dissolved in H_2O. The reaction was allowed to preceed overnight with gentle shaking. The reaction was then purified using RP-HPLC (A= 10% ACN in 50 mM TEAA buffer, B= 100% ACN) and fractions containing TA-SSA were freeze dried. Finally, the product was desalted using a NAP-10 DNA purification column (Cytiva). LC-MS analysis of TA-SSA confirmed the mass.

The modified sense strand was annealed with the antisense (AS) to form the final product: mono-TA-siRNA. In PCR tubes, oligos were mixed in equimolar ratrios (300-400 µM, 1x PBS), briefly heated to 90 °C for 5 mins and then incubated for 1 hour at 37 °C.

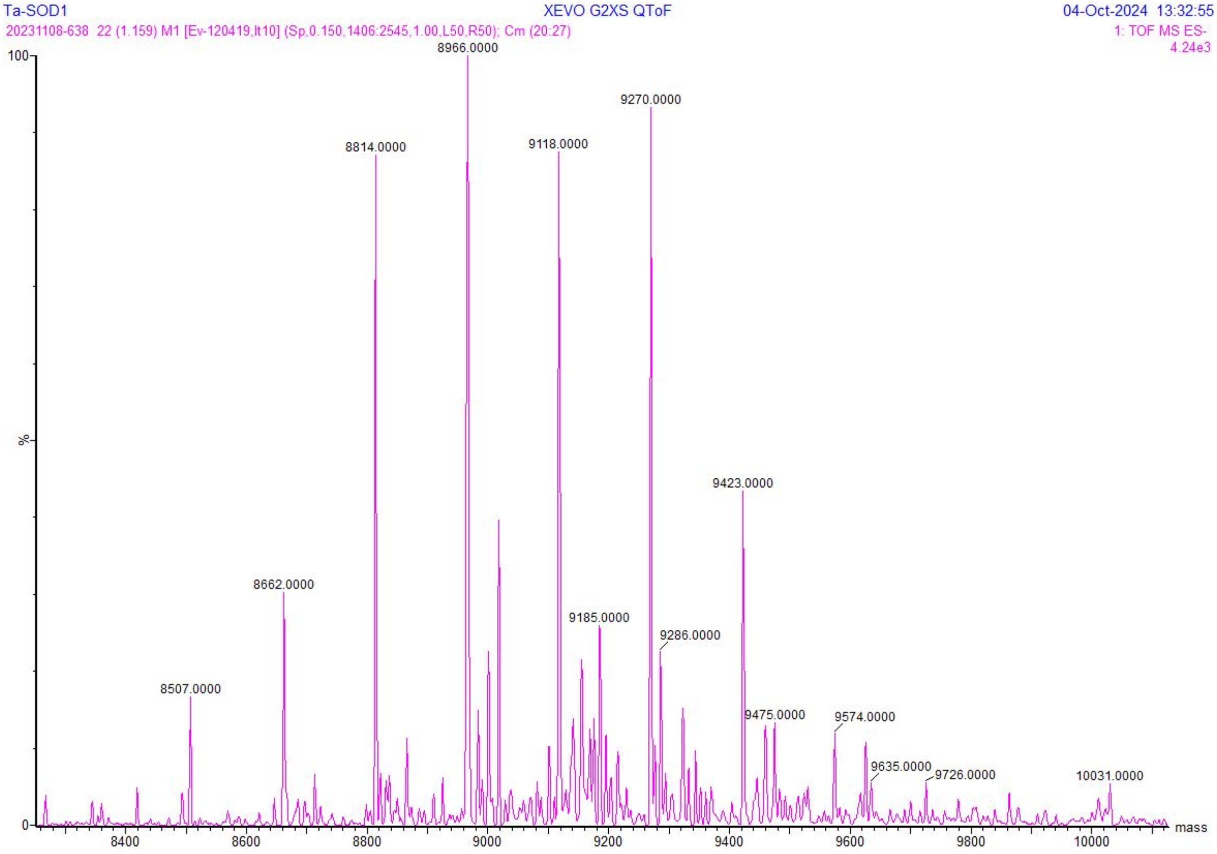

Figure S3. Deconvoluted mass specrum of TA-SSA

4.2 bis-TA-siRNA

In a 2mL eppendorf, 4 equiv. of TA-N_3, dissolved in H_2O, were added to 1 equiv. of SSB dissolved in H_2O. The reaction was allowed to preceed overnight with gentle shaking. The reaction was then purified using RP-HPLC (A= 10% ACN in 50 mM TEAA buffer, B= 100% ACN) and fractions containing TA-SSB-TA were freeze dried. Finally, the TA-SSB-TA was desalted using a NAP-10 DNA purification column (Cytiva). LC-MS analysis of product confirmed mass.

The modified sense strand was annealed with the antisense (AS) to form the final product, bis-TA-siRNA, using the same protocol described previously.

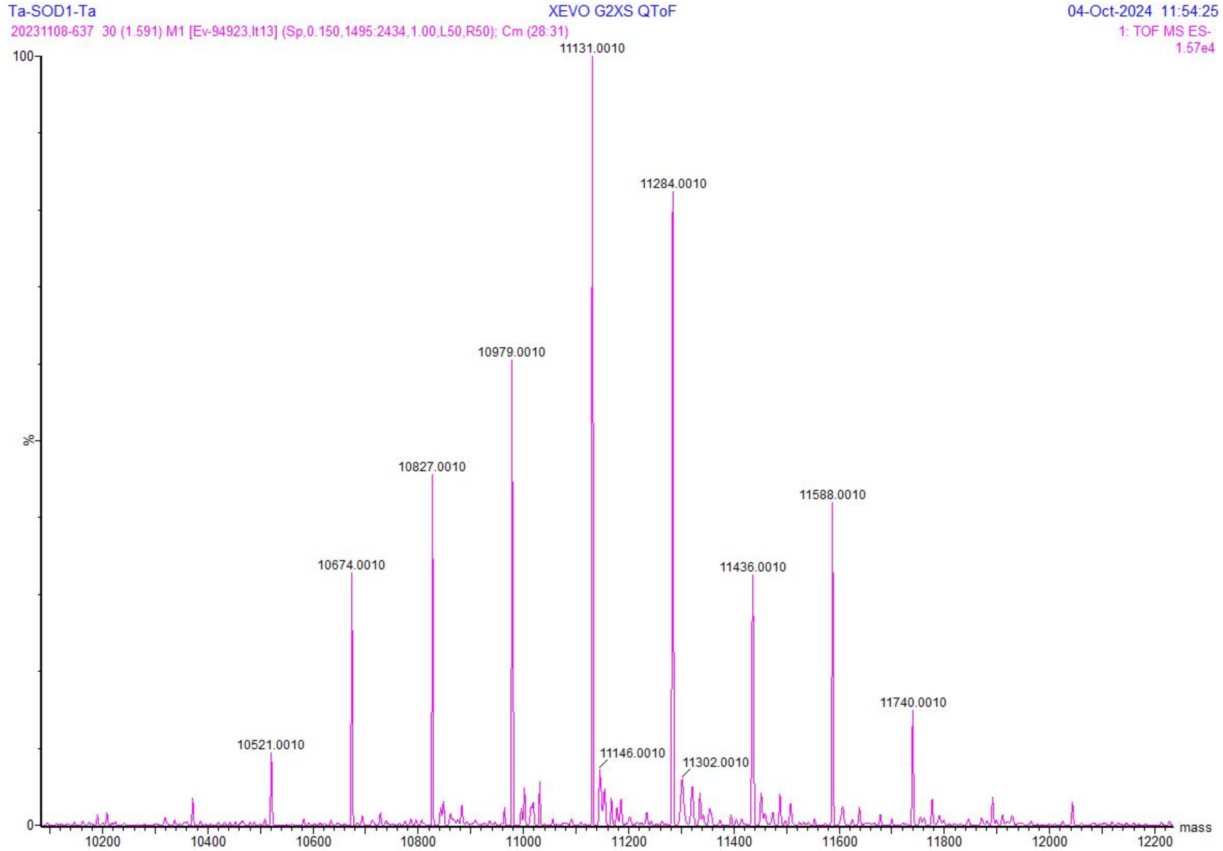

Figure S4. Deconvoluted mass specrum of TA-SSB-TA

4.3 tri-TA-siRNA

To generate a sense strand containing 3 BCN groups, 1 equiv. of SSB (dissolved in pH 8.8 carbonate buffer) was mixed with 1.5 equiv. of NHS-C5-bis-PEG3-BCN (ConjuProbe, dissolved in 100 ul DMF). The reaction was mixed at ambient temperature for 2 hours. The reaction was then purified using RP-HPLC (A= 10% ACN in 50 mM TEAA buffer, B= 100% ACN) and fractions containing SSC-bis-BCN were freeze dried. LC-MS analysis of product confirmed the mass.

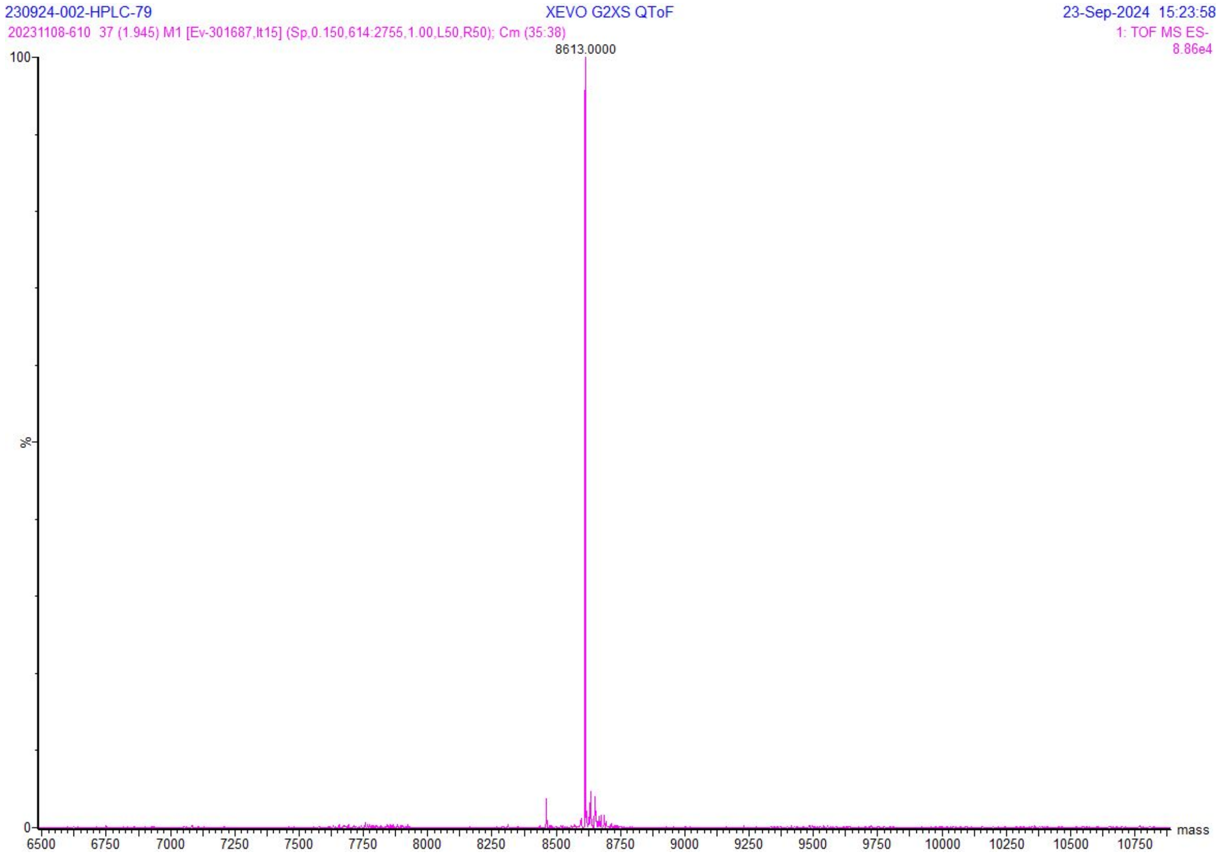

Figure S5. Deconvoluted mass specrum of SSC-bis-BCN

Next, in a 2mL eppendorf, 6 equiv. of TA-N_3, dissolved in H_2O, were added to 1 equiv. of BCN-Sod1-bis-BCN sense strand, also dissolved in H_2O. The reaction was allowed to preceed overnight with gentle shaking. The reaction was then purified using RP-HPLC (A= 10% ACN in 50 mM TEAA buffer, B= 100% ACN) and fractions containing TA-Sod1-bisTA were freeze dried. Finally, TA-Sod1-bisTA was desalted using a NAP-10 DNA purification column (Cytiva). LC-MS analysis of TA-Sod1-bisTA confirmed the mass.

The modified sense strand was annealed with the antisense (AS) to form the final product, tri-TA-siRNA, using the same protocol described previously.

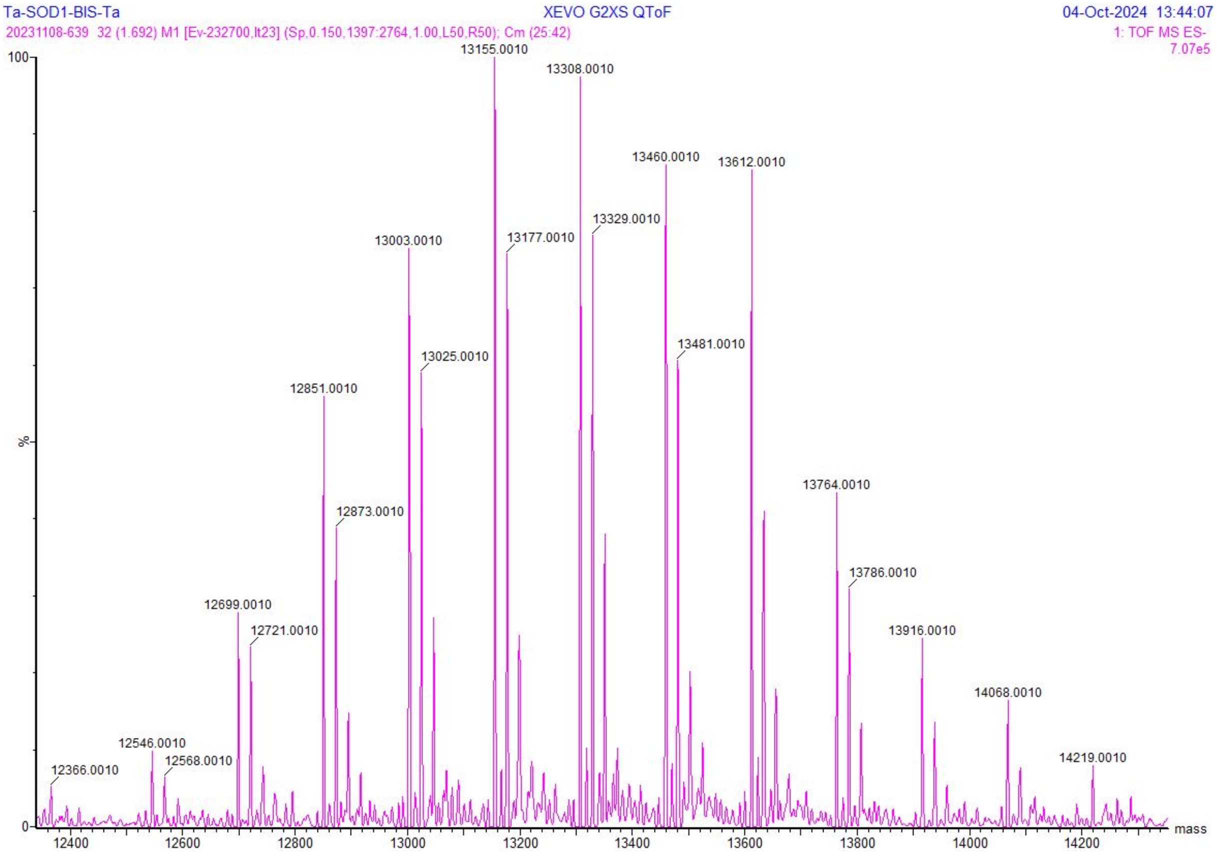

Figure S6. Deconvoluted mass specrum of TA-SSC-bis-TA

Synthesis of heterobifunctional TA-modified siRNA conjugates

4.4 siRNA-peg4-C16

In a 2 mL eppendorf, 2 equiv. of palmitic acid-PEG4-NHS ester (Broadpharm, DMSO) was added to 1 equiv. of SSC dissolved in pH 8.8 carbonate buffer. The reaction was incubated at 37 degrees with agitation on a table top shaker for 1 hour. Excess palmitic acid was removed by extraction with 3x washes of ethylacetate. The reaction was then purified using RP-HPLC (A= 10% ACN in 50 mM TEAA buffer, B= 100% ACN). Fractions containing SSC-peg4-C16 were collected and freeze dried. LC-MS analysis of SSC-peg4-C16 confirmed the mass.

The modified sense strand was annealed with the antisense (AS) to form the final product, siRNA-peg4-C16, using the same protocol described previously.

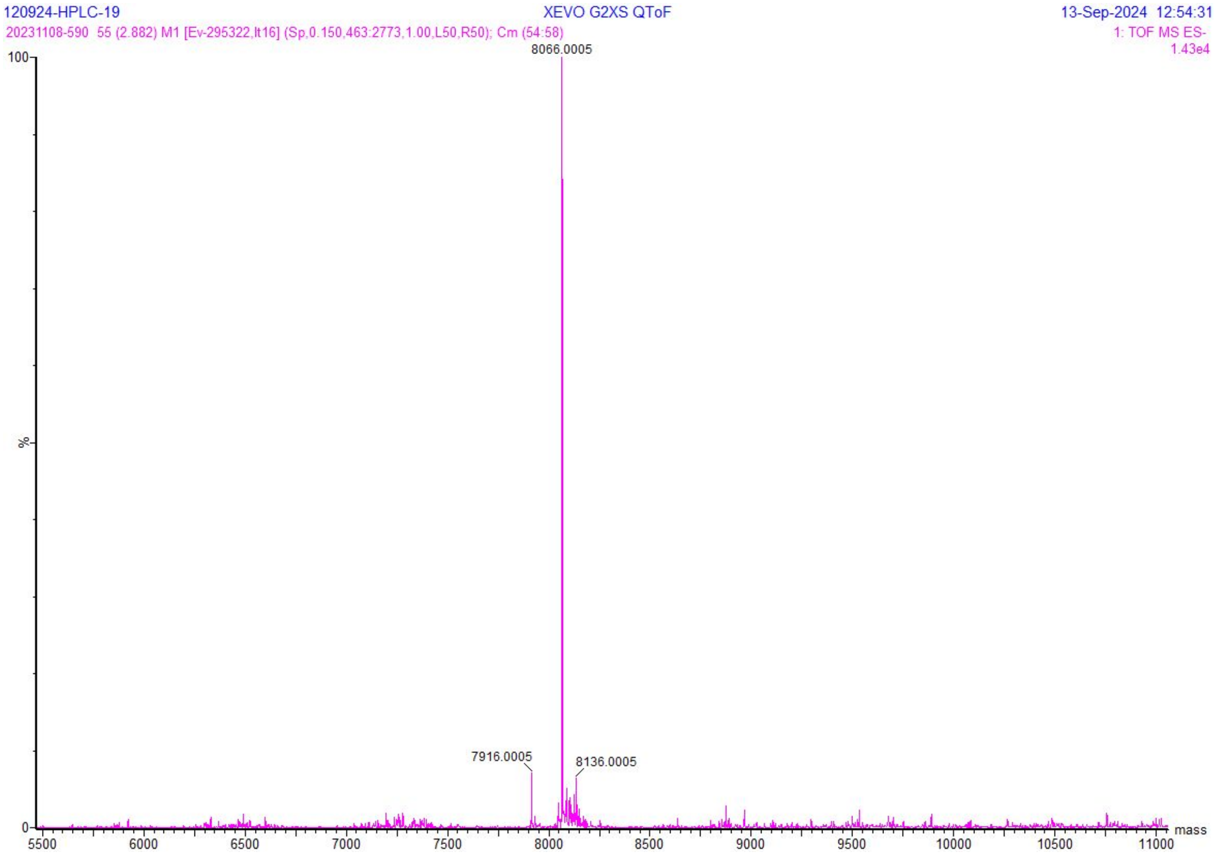

Figure S7. Deconvoluted mass specrum of SSC-peg4-C16

4.5 TA-siRNA-peg4-C16

In a 2 mL eppendorf tube, 2 equiv. of TA-N_3, dissolved in H_2O, were added to 1 equiv. of SSC-peg4-C16 dissolved in H_2O. The reaction was allowed to preceed overnight with gentle shaking. The reaction was then purified using RP-HPLC (A= 10% ACN in 50 mM TEAA buffer, B= 100% ACN) and fractions containing TA-SSC-peg4-C16 were freeze dried. Finally, TA-SSC-peg4-C16 was desalted using a NAP-10 DNA purification column (Cytiva). LC-MS analysis of TA-SSC-peg4-C16 confirmed the mass.

The modified sense strand was annealed with the antisense (AS) to form the final product, siRNA-peg4-C16, using the same protocol described previously.

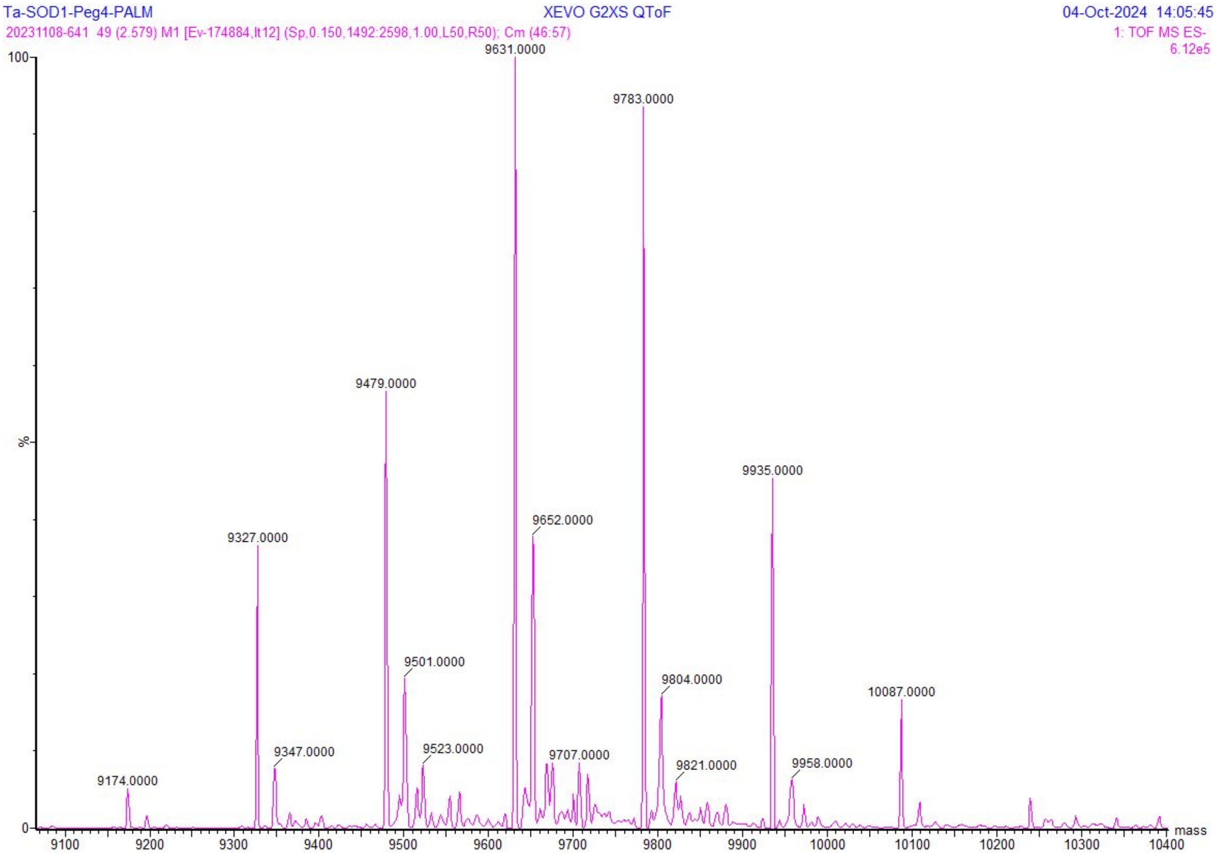

Figure S8. Deconvoluted mass specrum of TA-SSC-peg4-C16

4.6 siRNA-AlbuTag

Synthesis of AlbuTag (N^6-4-(4-iodophenyl)butanoyl-lysine)

In a flask, 1.2 equiv. of CDI was added to 1 equiv. of 4-(-p-iodophenyl)butanoic acid in dry DMF and allow to stir at room temperature for 45 mins. To this solution, 1.5 equiv. of Fmoc-Lys-OH-HCl (dissolved in DMSO) was added and the reaction was stired at room temperature over night. The reaction was quenched with water and then purified using RP-HPLC (A= 10% ACN in 50 mM TEAA buffer, B= 100% ACN). Fractions containing product were collected and freeze dried. Deprotection of the Fmoc group was performed using 20% pipiridine in DMF with stirring for 1 hour. Water was added and the mixture transferred to a seporatory funnel for extraction with 3x washes of ethylacetate. The aqueous phase was then freeze dried. LC-MS analysis of product confirmed mass.

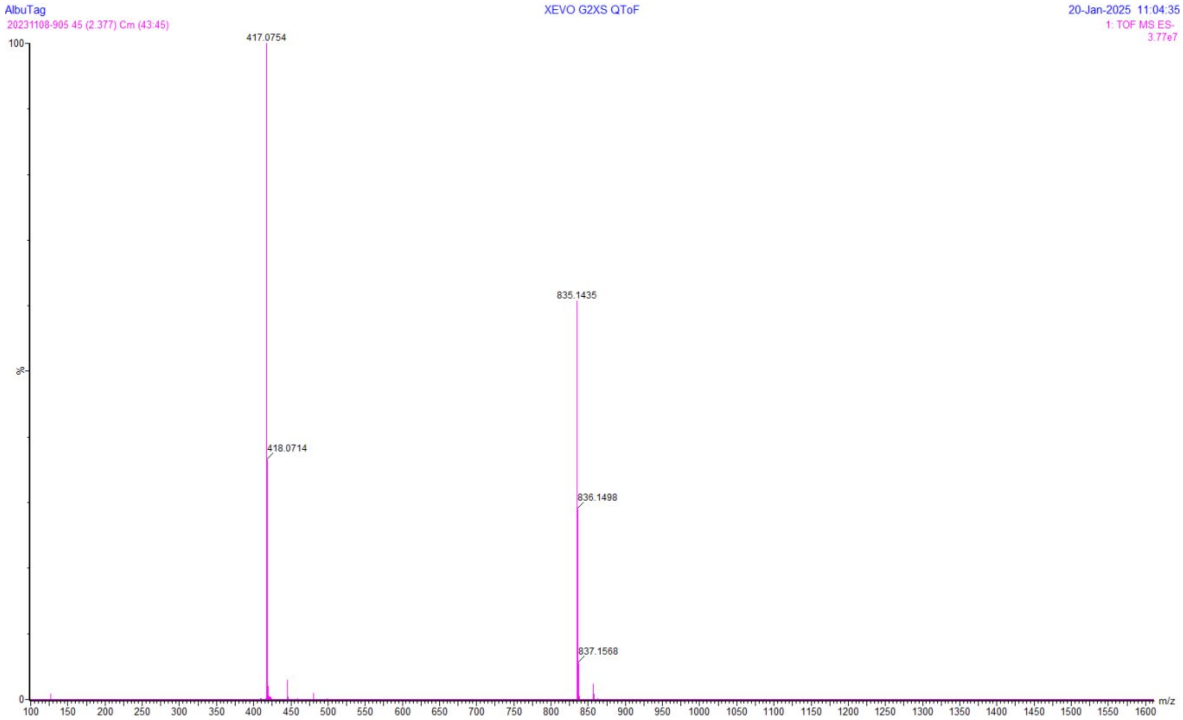

Figure S9. Mass specrum of AlbuTag

Next, in a 2 mL eppendorf, 1 equiv. of bis-peg2-NHS ester (Broadpharm, DMF) was added to 1 equiv. of AlbuTag (DMF) and 1 equiv. of SSC dissolved in pH 8.8 carbonate buffer. The reaction was incubated at ambient temperature with agitation on a table top shaker for 1 hour. The reaction was then directly purified using RP-HPLC (A= 10% ACN in 50 mM TEAA buffer, B= 100% ACN). Fractions containing SSC-Albutag were collected and freeze dried. Finally, SSC-AlbuTag was desalted using a NAP-10 DNA purification column (Cytiva). LC-MS analysis of SSC-AlbuTag confirmed the mass.

The modified sense strand was annealed with the antisense (AS) to form the final product, siRNA-Albutag, using the same protocol described previously.

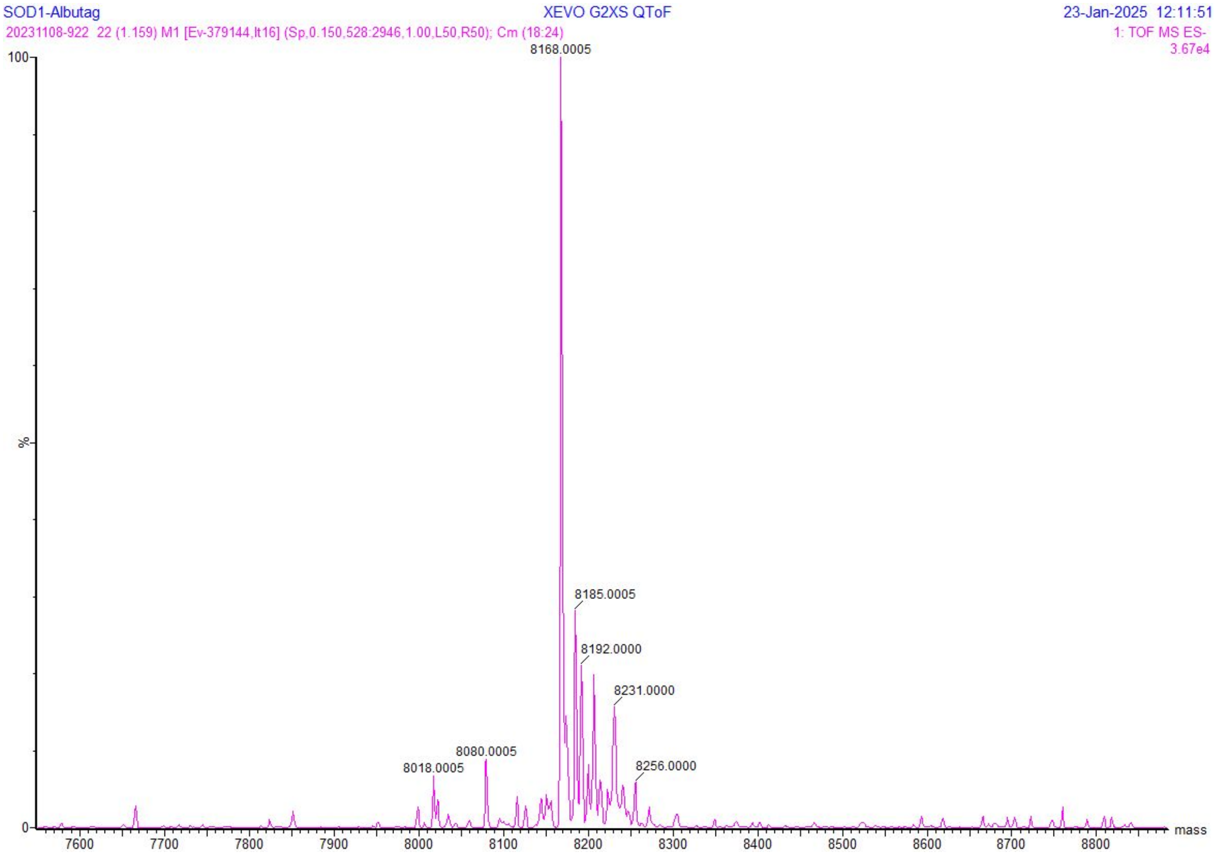

Figure S10. Deconvoluted mass specrum of SSC-AlbuTag

4.7 TA-siRNA-AlbuTag

In a 2 mL eppendorf tube, 2 equiv. of TA-N_3, dissolved in H_2O, were added to 1 equiv. of SSC-AlbuTag dissolved in H_2O. The reaction was allowed to preceed overnight with gentle shaking. The reaction was then purified using RP-HPLC (A= 10% ACN in 50 mM TEAA buffer, B= 100% ACN) and fractions containing TA-SSC-AlbuTag were freeze dried. Finally, TA-SSC-AlbuTag was desalted using a NAP-10 DNA purification column (Cytiva). LC-MS analysis of TA-SSC-AlbuTag confirmed the mass.

The modified sense strand was annealed with the antisense (AS) to form the final product, TA-SSC-AlbuTag, using the same protocol described previously.

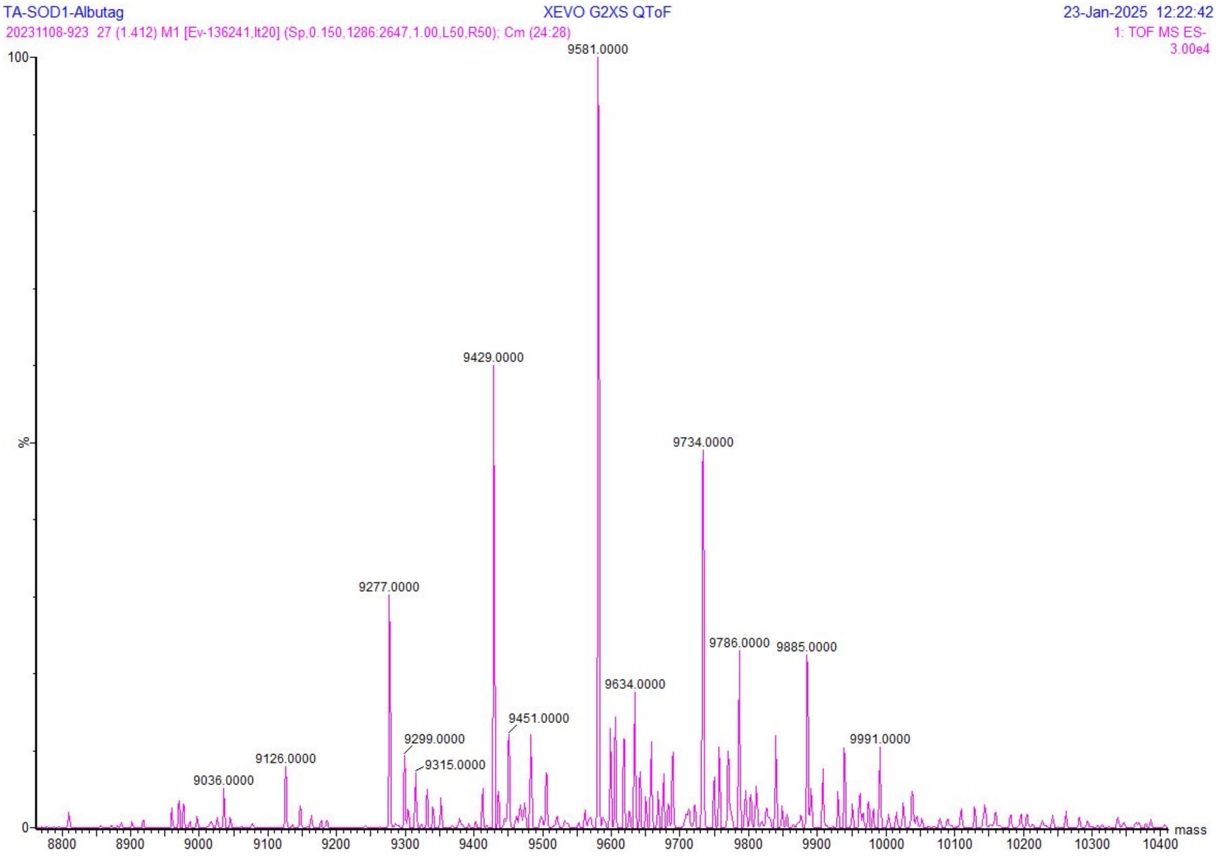

Figure S11. Deconvoluted mass specrum of TA-SSC-AlbuTag

4.8 LC-MS method

An ACQUITY UPLC BEH C18 (1.7 µm) 2.1 x 50 mm column (p/n 186002350) was employed to analyse cojugated oligonucleotides using mobile phase of water (A) and acetonitrile (B), both containing 5 mM ammonium acetate. Samples were injected (10 µl) onto the column (heated to 60 °C) and separation was achieved using a flowrate of 0.8 mL/min and a gradient of 10 to 90% B over 8 minuutes. Mass detection was performed using the Xevo™ G2XS QToF mass spectrometer in negative ionization mode.

5. DLS of TA conjugates

Dynamic light scattering (DLS) measurements were performed using a DynaPro ZetaStar instrument (Wyatt Technology) operating in backscattering mode. Samples of siRNA conjugate were prepared in phosphate-buffered saline (PBS) at a concentration of 150 µM and filtered where appropriate to minimize particulate contamination. Measurements were conducted at a controlled temperature of 25 °C following a brief equilibration period. For each sample, data were acquired as a series of 10 consecutive runs with an acquisition time of 10 s per run. This set of measurements was repeated three times to ensure reproducibility.

In DLS measurements of siRNA in PBS, the intensity-weighted size distribution is disproportionately influenced by a small population of large scatterers (>100 nm), due to the strong (d^6) dependence of scattering intensity. These species are attributed to trace aggregates or extrinsic contaminants (e.g., dust or buffer-derived particulates) and are not considered representative of the siRNA population, which is expected to have a hydrodynamic diameter in the 5–10 nm range. To better reflect the size distribution of the predominant siRNA species, the data are therefore presented as a number-weighted distribution.

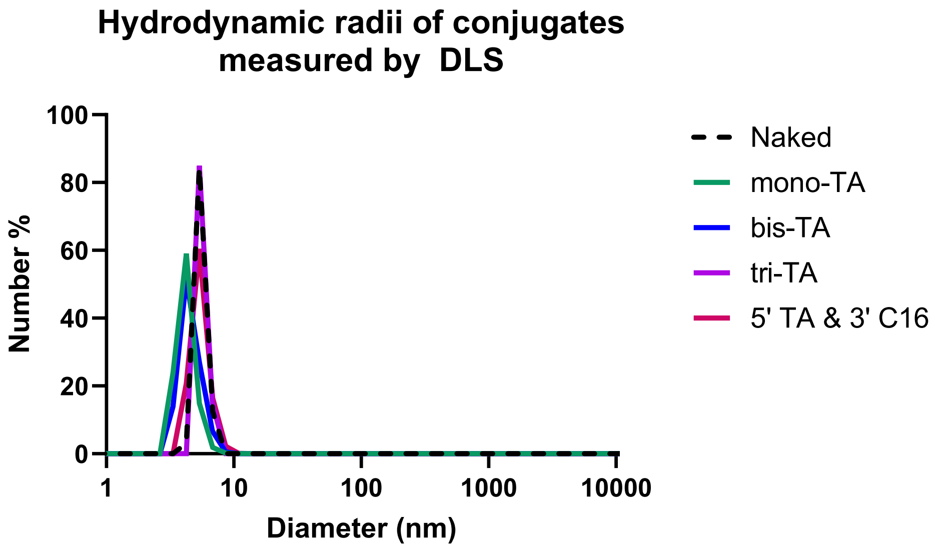

Figure S12. Dynamic Light Scattering (DLS) measurements of TA-conjugates.

6. In vitro evaluation of siRNA conjugates

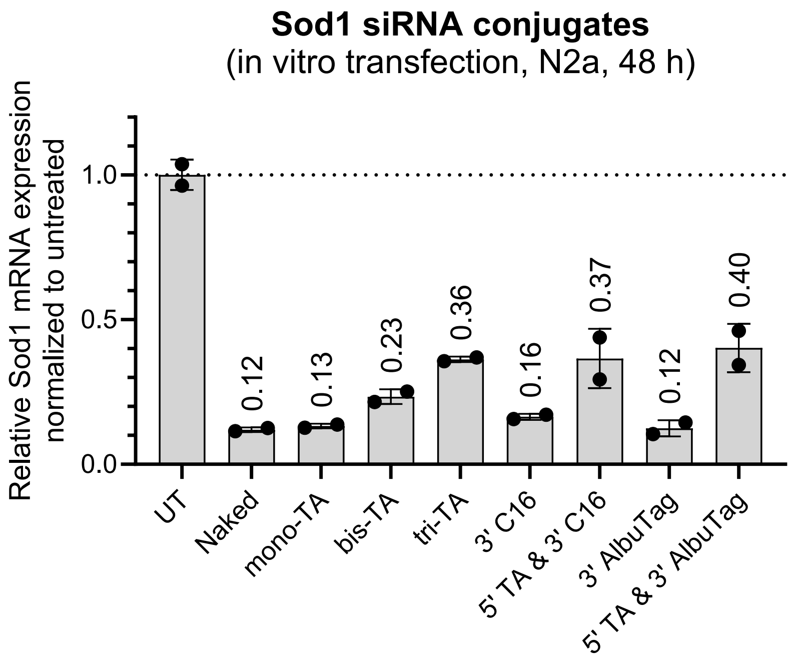

Figure S13. In vitro evaluation of siRNA conjugates.

Neuro-2a cells were transfected with 1 pmol/well of the indicated siRNA conjugates using RNAiMAX and analyzed 48 h later. Sod1 mRNA levels were quantified by TaqMan qPCR and normalized to Gapdh, relative to the untreated control.

7. Animal experiments

Animal experiments were approved by the Swedish Animal Ethics Committee in Linköping, Sweden (permit number 13849-2020; 14772-2023) under the supervision of the Swedish Board of Agriculture (Jordbruksverket) and conducted in accordance with national legislation and EU Directive 2010/63/EU for animal experimentation. Experimental procedures were designed to minimize animal suffering and the number of animals used. Animals were euthanized using approved humane methods in accordance with institutional and national guidelines.

NMRI mice (female, 4-5 weeks old or 20-25 g) were obtained from Janvier Labs. Animals were housed at the Preclinical Laboratory (PKL), Novum, Karolinska University Hospital, Huddinge, under specific pathogen-free conditions compliant with national animal welfare legislation. Animals were acclimatized for at least 7 days following arrival from the supplier. Mice were group-housed in individually ventilated cages (IVC; maximum five adult animals per cage) with wood-chip bedding and environmental enrichment including nesting material, gnawing sticks, and shelters. Animals had ad libitum access to food and water and were maintained at an ambient temperature of 20–22 °C, 45–55% humidity, and a 12 h light/dark cycle. Animals were monitored daily by trained animal care staff with veterinary supervision available when required.

7.1 In vivo experiments and knockdown analysis by qPCR

Mice were tail-vein injected with siRNAs at 600 nmol/kg. Seven days post-injection mice were euthanized, and tissues were harvested and stored at -80C until further processing.

Tissues were homogenized in TRI Reagent® (T9424, Sigma-Aldrich) on TissueLyser II (QIAGEN) with stainless steel beads (heart) or on gentleMACS™ Dissociator (Miltenyi Biotec) using program RNA_02_01 (liver). Total RNA was extracted according to manufacturer's instructions (TRI Reagent®) and reverse-transcribed with High Capacity cDNA Reverse Transcription Kit (43-688-13, Applied Biosystems™). qPCR was performed on a CFX Opus 96 Real-Time PCR Instrument (Bio-Rad) using TaqMan™ Fast Advanced Master Mix (4444557, Applied Biosystems™) and TaqMan® Gene Expression Assays (Sod1 = Mm01344233_g1, Gapdh = Mm99999915_g1). Following the ΔΔCt method, Sod1 expression was analyzed relative to Gapdh, and normalized to the PBS-treated control group.
